# Association between sleep and periodontal disease in adults—an umbrella review

**DOI:** 10.3389/froh.2026.1761243

**Published:** 2026-03-12

**Authors:** Keerthana Rajeev, Chandrashekar Janakiram, Vijay Kumar S

**Affiliations:** Amrita School of Dentistry, Amrita Vishwa Vidyapeetham University, Kochi, India

**Keywords:** obstructive sleep apnoea, periodontal disease, periodontitis, sleep duration, sleep quality

## Abstract

**Introduction:**

Sleep disturbances and periodontal disease are common chronic conditions linked through systemic inflammation; however, their association remains unclear despite the availability of several secondary studies.

**Methods:**

An umbrella review was conducted to synthesise evidence from published systematic reviews and meta-analyses that examined the relationship between sleep duration and sleep disorders, including obstructive sleep apnoea, and periodontal disease in adults. Major electronic databases were searched from inception to March 2024 without language restrictions. The methodological quality of the included reviews, overlap of primary studies, and certainty of evidence were systematically assessed.

**Results:**

Thirteen systematic reviews were included, of which seven contained meta-analyses, representing fifty-two unique primary studies. Overall, poor sleep and sleep disorders were associated with a higher likelihood of periodontal disease; however, the magnitude of the effect varied considerably and substantial heterogeneity was observed. There was a high degree of overlap among primary studies, and the certainty of the evidence was low, largely due to observational study designs, risk of bias, and residual confounding.

**Discussion:**

This umbrella review indicates a positive but inconsistent association between sleep disturbances and periodontal disease. The findings emphasise important limitations in the current evidence base and highlight the need for well-designed prospective studies with standardised assessments to clarify causal pathways and underlying mechanisms.

**Systematic Review Registration:**

https://www.crd.york.ac.uk/PROSPERO/view/CRD42024511133, PROSPERO CRD42024511133.

## 1 Introduction

Stress and insufficient sleep have emerged as major public health challenges in modern societies. Sleep is a complex biological process essential for physical, cognitive, metabolic, and immunological functioning across all demographic groups (1). However, lifestyle changes, extended work hours, increased screen time, and competing responsibilities have significantly disrupted sleep patterns in recent decades. According to the Centers for Disease Control and Prevention (CDC), one in three American adults does not obtain adequate sleep, and an estimated 50–70 million individuals experience chronic sleep disturbances (2).Although often overlooked, healthy sleep—characterized by adequate duration, good quality, regularity, and appropriate timing (3). —is critical for maintaining systemic homeostasis. Sleep deprivation may be acute or chronic (4), and has been associated with hormonal dysregulation, including reduced testosterone and elevated cortisol levels. Disrupted sleep impairs attention, mood, behaviour, and productivity and increases the risk of obesity, diabetes, cardiovascular disease, stroke, and hypertension (5).

Obstructive sleep apnea (OSA) is the most common sleep-related breathing disorder, affecting nearly one billion people worldwide and remaining substantially underdiagnosed (6). It is characterized by repeated episodes of reduced or absent airflow despite ongoing respiratory effort (7). Major risk factors include male sex, aging, and obesity, with additional contributors such as craniofacial anatomical variations, supine sleeping position, alcohol use, smoking, and endocrine or neurological disorders (8). Clinically, OSA presents with loud snoring, witnessed apneas, and excessive daytime sleepiness; more than 85% of individuals with clinically significant OSA remain undiagnosed (9). Beyond its respiratory manifestations, OSA is increasingly recognized as a systemic inflammatory condition, with intermittent hypoxia triggering oxidative stress, sympathetic activation, and dysregulation of inflammatory cytokines. These pathophysiological processes may plausibly influence periodontal health.

Periodontitis is a chronic inflammatory disease of the tooth-supporting structures and a major cause of tooth loss globally (10). Nearly 19% of the global population is affected by severe periodontitis, representing about one billion individuals (11). Clinical features include gingival bleeding and swelling, tooth mobility, dentinal hypersensitivity, and halitosis (12). Evidence suggests a biological link between sleep disturbances and periodontal disease through shared immune-inflammatory pathways. Sleep disturbances alter inflammatory biomarkers such as C-reactive protein (CRP), tumour necrosis factor-α (TNF-α), and interleukin-6 (IL-6), contributing to periodontal tissue breakdown (13, 14).

A preliminary search identified several systematic reviews examining associations between sleep duration, sleep quality, and OSA with periodontal disease; however, findings remain inconsistent. Muniz et al. (13) reported conflicting associations between self-reported sleep duration and periodontitis and were unable to conduct a meta-analysis due to substantial heterogeneity in exposure measures such as the Pittsburgh Sleep Quality Index (PSQI). Lembo et al. (15) found generally positive associations between OSA and periodontitis, yet variability in exposure definitions (Apnea–Hypopnea Index, oxygen desaturation indices) and periodontal outcomes [clinical attachment loss (CAL), probing pocket depth (PPD), alveolar bone loss (ABL)] prevented meta-analysis.

A meta-analysis by Al-Jewair et al. (16) found a significant association (OR 1.65; 95% CI 1.11–2.46) between periodontal disease and OSA despite low quality evidence. Similarly, Khodadadi et al. (17) reported a strong association between OSA and mild-to-moderate periodontitis (OR 2.17; 95% CI 1.66–2.83), but no significant association with severe disease. Conversely, reviews by Wu, Malaiappan, and Schmidlin questioned the strength or consistency of this relationship (18–20). Variability in outcome definitions, inconsistent Apnea–Hypopnea Index thresholds, non-standardized periodontal assessment methods, incomplete adjustment for confounders, and overlapping primary studies contribute to conflicting conclusions.

Given the interconnected nature of sleep, immunity, and chronic inflammation, elucidating the relationship between sleep and periodontal disease is of substantial clinical and public health relevance. Poor sleep has cascading effects on systemic immunity, autonomic balance, and metabolic function, all of which may influence susceptibility to periodontal breakdown. Likewise, chronic periodontal inflammation may itself contribute to sleep disturbances through pain, systemic inflammation, or comorbidities. This potential bidirectional relationship underscores the need for integrated, interdisciplinary care approaches involving dental and medical practitioners.

While evidence suggests an association between sleep disturbances and periodontal disease, methodological limitations and inconsistent findings highlight the need for a comprehensive umbrella review that synthesizes and appraises the highest level of available evidence. Such a review can evaluate methodological rigor, quantify overlap of primary studies, determine the certainty of evidence, and guide future research. High-quality longitudinal and interventional studies with standardized sleep and periodontal assessments are needed to clarify mechanisms, establish causality, and inform evidence-based preventive and therapeutic strategies.

### 1.1 Review question

What is the association between sleep and the occurrence of periodontal disease in adults?

### 1.2 Inclusion criteria

#### 1.2.1 Population

This umbrella review included systematic reviews that examined adults aged ≥18 years. No restrictions were applied regarding sex, ethnicity, socioeconomic status, geographic location, or general health status of the participants.

#### 1.2.2 Exposure

Systematic reviews that assessed sleep or sleep-related conditions in adults using objective or subjective measures were eligible. Accepted assessments included:

- Pittsburgh Sleep Quality Index (PSQI): a 19-item questionnaire generating seven component scores (0–3 each), summed to a global score ranging from 0 to 21. Higher scores denote poorer sleep quality (21).
- Epworth Sleepiness Scale (ESS): measures daytime sleepiness through eight items scored 0–3 (22).
- Stanford Sleepiness Scale (SSS): a single-item self-report rating of current sleepiness from 1 (highly alert) to 7 (unable to stay awake) (23).
- Berlin Questionnaire: identifies risk for OSA based on snoring behaviours, daytime sleepiness, and clinical risk factors.
- STOP or STOP-BANG questionnaire: screens for sleep apnea; STOP-BANG adds BMI, age, neck circumference, and gender to improve sensitivity (24):
- Apnoea Risk Evaluation System [ARES]
- Sleep diaries: participant-reported logs detailing sleep–wake timing and perceived sleep quality (25).

Reviews that used validated questionnaires, sleep duration reports, sleep quality indices, or physiological sleep parameters were eligible.

#### 1.2.3 Outcome

Eligible systematic reviews must have reported periodontal disease as a primary outcome. Periodontal disease was characterized using standardized clinical parameters, including:

- Clinical Attachment Loss (CAL)
- Probing Pocket Depth (PPD)
- Alveolar Bone Loss (ABL)
- Gingival Recession
- Oral hygiene indices (e.g., Plaque Index, Gingival Index)

These parameters reflect the severity and extent of periodontal destruction and are routinely applied in periodontal diagnosis, progression assessment, and treatment evaluation.

#### 1.2.4 Types of studies

This umbrella review included systematic reviews with or without meta-analysis. A paper was considered a systematic review if it satisfied the following criteria:

1. A clearly stated PEO framework (Population, Exposure, Outcome) articulated as a research question or objective.
2. A transparent and reproducible search strategy describing databases searched, search terms, and limits applied.
3. Explicit inclusion criteria specifying eligible study designs, populations, exposures, or outcomes.
4. Critical appraisal of included primary studies using a recognized methodological quality assessment tool.

These criteria ensured methodological rigor and transparency across the reviews included in this umbrella synthesis.

## 2 Methods

The proposed review was meticulously carried out in accordance with the JBI methodology specifically designed for umbrella reviews (26). This methodology ensures a systematic and rigorous approach to synthesizing evidence from multiple systematic reviews on a particular topic. The protocol detailing the review process has been formally registered with PROSPERO, an international database of prospectively registered systematic reviews in health and social care (CRD42024511133).

Furthermore, adherence to the Preferred Reporting Items for Systematic Reviews and Meta-Analyses (PRISMA) guidelines was integral in structuring the manuscript (27). These guidelines provide a standardized framework for transparent reporting of systematic reviews, ensuring clarity and reproducibility.

### 2.1 Search strategy

The search strategy was designed to comprehensively identify systematic reviews, both published and unpublished, that examined the relationship between sleep and periodontal disease in adults, with or without meta-analysis. A preliminary search was conducted in MEDLINE (PubMed), the Cochrane Database of Systematic Reviews, Scopus, Web of Science, Embase, and CINAHL (EBSCOhost). This initial search utilized text words found in titles and abstracts, and index terms specific to each database (Supplementary Appendix SI).

The search strategy was adapted for each database and reference lists of selected reviews were screened to identify additional relevant articles. Reviews published in any language were considered from the inception of each database up to the present. DeepL software (DeepL SE, Cologne, Germany) was employed to translate non-English and Malayalam publications.

Google Scholar was manually searched up to the first 10 pages to identify secondary resources. Review registers, including the Cochrane Database of Systematic Reviews, PROSPERO, and the JBI Systematic Review Register, were also consulted to capture ongoing or recently completed systematic reviews.

### 2.2 Study selection

After collating citations and uploading them into JBI SUMARI (28), duplicates were removed. Two independent reviewers (KR and CJ) conducted a pilot-tested screening of titles and abstracts. Potentially relevant citations underwent full-text review by KR and SV, with disagreements resolved through consensus or by a third reviewer (CJ).

The PRISMA flow diagram (27) was used to document the screening and selection process. In cases where systematic reviews included overlapping primary studies, both reviews were retained. A citation matrix was created to visualize overlap, and the corrected covered area (CCA) index was calculated (29). Reviewers were not blinded to journal titles, authors, or affiliations, ensuring transparency and consistency.

### 2.3 Assessment of methodological quality

All included systematic reviews were critically appraised by two independent reviewers (KR and SV) using the JBI critical appraisal tool for systematic reviews and research syntheses (30). Discrepancies were resolved through discussion or consultation with the third reviewer (CJ). The results of the appraisal were presented both narratively and in tables, documenting strengths and limitations. Despite differences in methodological quality, all reviews underwent data extraction where feasible.

### 2.4 Data extraction

Data extraction was performed by two independent reviewers using a customized and pilot-tested JBI data extraction tool (Table 1). Both reviewers completed extraction for one review initially and refined the tool based on usability and completeness.

A third author reviewed the completed extraction forms to ensure accuracy and completeness. The tool was iteratively refined throughout the review to ensure comprehensive capture of all relevant data. This structured and rigorous approach to data extraction ensured consistency and reliability in synthesizing findings pertaining to the association between sleep and periodontal disease.

### 2.5 Data synthesis

In this umbrella review, all data extracted from the included systematic reviews were systematically tabulated and accompanied by a narrative synthesis to address the review's objectives and research questions. For each systematic review, key characteristics were documented, including the number of primary studies contributing to each outcome, total sample size, and reported heterogeneity estimates (31).

The overlap of primary studies across systematic reviews was examined using a citation matrix, and the extent of duplication was quantified to ensure transparency regarding the distribution of evidence. Although results from existing meta-analyses within the included reviews were summarized, no re-analysis or re-pooling of data was undertaken due to substantial methodological variability across reviews.

For systematic reviews that did not conduct a meta-analysis, findings were synthesized narratively, emphasizing patterns, consistencies, and divergences in reported associations. Where available, pooled effect sizes such as odds ratios, risk ratios, hazard ratios, or standardized mean differences were extracted and reported alongside the corresponding assessments of methodological quality.

To minimize publication bias, an extensive search strategy—including grey literature—was implemented to identify unpublished systematic reviews and reduce the likelihood of selective reporting. Associations between sleep parameters and periodontal disease were extracted either from pooled quantitative findings or from narrative summaries reported in the included reviews.

Quantitative results were categorized according to periodontal outcomes (e.g., CAL, PPD, ABL) and types of sleep assessments (e.g., sleep duration, sleep quality, OSA severity). These findings were organized into structured tables that presented effect estimates within and between exposure groups, enabling clear comparison across the evidence base. This structured synthesis approach enabled a comprehensive evaluation of the association between sleep and periodontal disease and supported robust, evidence-informed conclusions.

### 2.6 Assessment of publication overlap and corrected covered area (CCA) index

Publication overlap across the systematic reviews included in the umbrella review was quantified using the Corrected Covered Area (CCA). White indicated a slight degree of overlap (15%). To calculate the CCA, the “Graphical Representation of Overlap for OVErviews” (GROOVE) tool was used. The CCA index was determined using the formula:

CCA=(N–r)/(r×c–r)

where *N* represents the total number of times primary publications appeared across the reviews (including duplicate occurrences), r is the number of unique primary publications, and c is the number of systematic reviews included in the umbrella review.

An evidence matrix was constructed by one author (HD) documenting all primary studies included in each systematic review, with a second author (CJ) validating the extraction for accuracy. This approach provided transparency regarding study duplication and informed the interpretation of synthesized evidence.

### 2.7 Assessing certainty in the findings

The Grading of Recommendations, Assessment, Development and Evaluation (GRADE) approach was applied to assess the certainty of evidence in this umbrella review (32). Using GRADEpro 2021 (McMaster University, Ontario, Canada), a Summary of Findings table was generated to present the associations between periodontal disease (PD) and obstructive sleep apnea (OSA). The certainty of evidence was evaluated using the following criteria:

1. Quality of primary studies—assessing risk of bias and methodological limitations.
2. Consistency of findings—examining the direction and magnitude of effects using heterogeneity values (*I*^2^): high (>60%), moderate (40–60%), or low (<40%)
3. Directness of evidence—determining applicability of included studies to the review question.
4. Precision of results—considering sample sizes and the number of contributing studies.
5. Publication bias—assessed qualitatively (32, 33)

When multiple systematic reviews presented meta-analyses for the same outcomes, selection for synthesis was based on criteria prioritizing lower heterogeneity (*I*^2^), a higher number of primary studies, and lower risk of bias. These reviews were summarized in a structured Summary of Findings table that reported intervention or exposure names, comparators, specific outcomes, effect estimates, and key interpretations.

To visually depict the strength of associations, a simple stoplight schema was used: green indicated strong association, yellow moderate evidence, and red weak or no association between periodontal disease and obstructive sleep apnea. This visualization facilitated clear communication and interpretation of evidence on the association between sleep and periodontal disease

## 3 Results

### 3.1 Study inclusion

**Figure 1:**
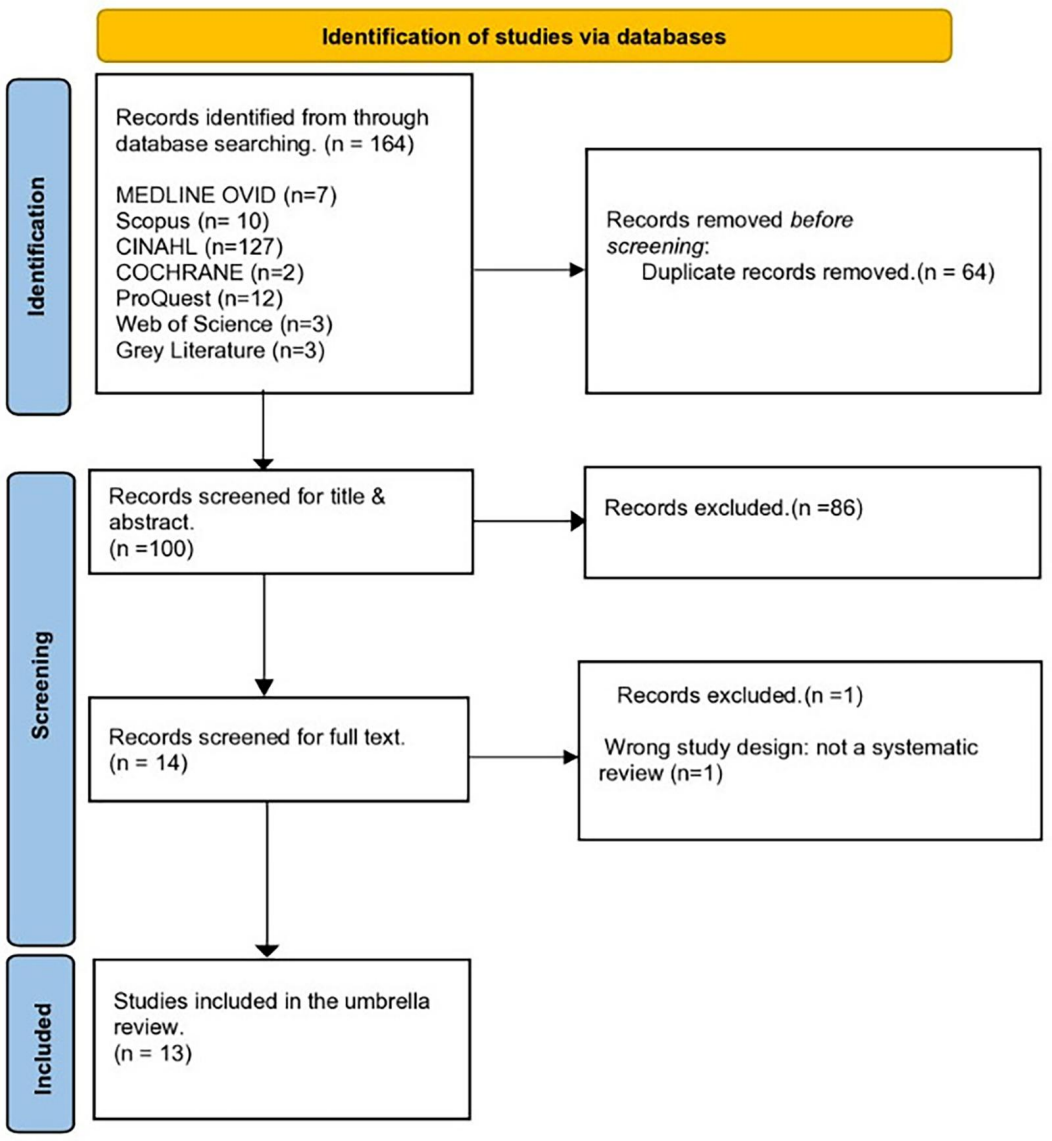
PRISMA flowchart of study selection and inclusion process.

The systematic review process began with a comprehensive search that initially identified 164 potentially relevant records across various databases. Following the removal of duplicates, a detailed screening of titles and abstracts narrowed down the selection to 100 records. These were further scrutinized, leading to the retrieval of 14 reviews for thorough evaluation against the inclusion criteria. From these, one review was excluded since it had a different study design. Ultimately, 13 systematic reviews were deemed suitable for inclusion in the umbrella review after meeting all predefined criteria (13, 15–20, 34–39). These reviews were critically appraised and their findings synthesized to address the overarching research questions. A detailed PRISMA flow diagram (Figure 1) visually represents the stages of screening, exclusion, and inclusion throughout this process, ensuring transparency and clarity in the review methodology. Supplementary Appendix SI complements this diagram by providing a summarized list of the excluded studies, along with specific reasons for their exclusion, thus enhancing the comprehensiveness and reliability of the review's findings

### 3.2 Characteristics of included reviews

The review encompassed an evaluation of 13 systematic reviews published between 1966 and 2024, of which seven included meta-analyses (16–18, 36–39). (Table 1) These reviews conducted comprehensive searches across multiple databases, prominently featuring MEDLINE (via PubMed), EMBASE, and the Cochrane Library, covering literature from 1966 to 2024. Most reviews involved a minimum of two database searches, with the exception of one (19). Predominantly, the primary studies were conducted in China (18, 36–38). The number of studies included in each systematic review varied, with a maximum of 20 studies reported in one review (13) and a minimum of five studies in another. The largest number of participants reported in a single review was 232,403 participants (13), while the smallest was 475 participants (34). Language restrictions were not applied to the reviews, all of which were published in English.

**Table 1:** Characteristics of included reviews.

Study ID	Country of review	Year of study	Number and name of data bases searched	Publication date ranges of the studies	Number and type of study design	Sample size	Assessment tool used to appraise the primary study and the rating of that quality	Exposure and its Assessment	Outcome and its Assessment	Method of synthesis	Assessment of publication bias	Conclusion
Lembo 2021 (15)	Italy	2021	4 Databases: PubMed, Scopus, LILACS Cochrane library	Jan 2009 to Sept 2020	10 studies: 5 case-control 5 cross-sectional	43,122	Case-control studies: Newcastle Ottawa Scale (NOS) Cross sectional studies: Adapted form of Newcastle Ottawa Scale (NOS)	Obstructive Sleep Apnoea: Polysomnography, Apnea Risk Evaluation System (ARES), Apnea- Hypopnea Index(AHI)	Periodontal Disease: Clinical Attachment Level (CAL), Pocket Depth (PD), Alveolar Bone Loss (ABL), Recession (REC) Bleeding on Probing (BoP), Periodontal Index (PI), Gingival Index (GI), or salivary cytokines	Narrative synthesis	Not assessed	There is low evidence of a possible association between obstructive sleep apnoea syndrome and periodontitis
Wu 2022 (18)	China	2022	3 Databases: PubMed, EMBASE, Cochrane libraries	1 Jan 1966 to 31 March 2021	6 studies: All cross-sectional	1,07,777	Agency for Healthcare Research and Quality (AHRQ) 0-3: low quality, 4-7: medium quality (4 studies), 8-11: high quality (2 studies)	Sleep duration: Self-reported	Periodontal disease: Clinical diagnoses, self-reported	Meta-analysis	Assessed	There is a link between PD and insufficient sleep duration. People who slept for an average <7 h had a 19% higher risk of developing PD.
Qun Zhou 2024 (36)	China	2024	4 Databases: PubMed, EMBASE, Web of Science, Cochrane library	Up to November 2022	11 studies: All Cross-sectional	70,923	Agency for Healthcare Research and Quality (AHRQ) 0-3: low quality 5–8: fair quality (3 articles) 9–11: high quality (8 articles)	Sleep duration: Self-reported questionnaires	Periodontitis: Clinical Attachment Loss(CAL), Community Periodontal Index(CPI)	Meta-analysis	Assessed	Neither short sleep duration nor long sleep duration was linked to periodontitis or severe periodontitis. However, a significant association was found when sleep duration ≤5 h.
Edoardo Bianchi 2024 (35)	Italy	2024	1 Database: MEDLINE/PubMed	2000 to December 2021	14 studies: 6 Case-control 8 Cross-sectional	43,773	Case-control studies - Newcastle Ottawa Scale (NOS) Cross sectional studies-adapted form of NOS Low: score>8, Medium: score 6–8, High Risk: score <6	Obstructive Sleep Apnoea: Polysomnography (PSG) conducted in a sleep laboratory or at home with portable devices (HST), Epworth Sleepiness Scale (ESS), Berlin questionnaire, STOP-BANG questionnaire	Periodontal Disease: Probing Depth (PPD), Clinical Attachment Level (CAL), Plaque Index (PI), Bleeding on Probing (POB)	Narrative synthesis	Not assessed	Some evidence of a plausible association between periodontitis and obstructive sleep apnoea.
Giorgio Bianchi 2022 (34)	France	2022	3 Databases: PubMed, EMBASE, Cochrane library	Up to 2022 April	8 Studies: All Cross-sectional	475	Adapted form of Newcastle Ottawa Scale (NOS) Score of 7 or more: good quality	Obstructive Sleep Apnoea: Apnea–Hypopnea Index (AHI)	Composition of oral microbiota: Salivary samples, Oral mucosal swabs, Subgingival plaque sample	Narrative synthesis	Not assessed	Obstructive Sleep Apnoea and related conditions (e.g: mouth breathing) may influence the composition of the oral microbiota.
Khodadadi 2022 (17)	Iran	2022	4 Databases: PubMed, EMBASE, Scopus, Web of Science	From inception till 31 Dec 2021	10 Observational studies(type-not specified)	30,994	Case-control studies: Newcastle Ottawa Scale (NOS): 0 to ≥6 points: high quality, 3–5 points: moderate quality, ≤2 points: low quality. Cross sectional studies: Adapted form of NOS: 0–10 ≥ 6 points: high quality, 3–5 points: moderate quality, ≤2 points = low quality)	Obstructive Sleep Apnoea: Polysomnography or a validated questionnaire.	Periodontitis 2017 Classification System of Periodontal and Peri-implant Diseases and Con­ditions or The 1999 Consensus Classification System of Periodontal Disease	Meta-Analysis	Assessed	Periodontitis is associated with mild-to-moderate obstructive sleep apnoea but not with severe obstructive sleep apnoea
Malaiappan 2019 (19)	India	2019	1 Database: PubMed	Not reported	5 Studies: 4 Cross-Sectional 1 Case-Control	1169	Cross sectional studies: Adapted form of Newcastle Ottawa Scale (NOS) Case-control Studies: Not mentioned	Sleep: Apnoea-Hypopnea Index, Daily sleep in hours, apnoea during sleeping, Presence of snoring, Insomnia and increased sleep apnoea probability	Periodontitis: Bleeding on brushing Gingival inflammation Clinical attachment loss(CAL)	Narrative synthesis	Not assessed	Due to the role played by various parameters, there is association between sleep and periodontitis
Al-Jewair 2015 (16)	UK	2015	7 Databases: PubMed, CINAHL, EMBASE, Cochrane Central Trial Registry, Cochrane Database of Systematic Reviews, Scopus, Embase SIGLE(For grey literature)	Until October 2014	6 studies: 3 case-control, 3 cross-sectional	30,130	Case-control studies: Newcastle Ottawa Scale (NOS) Cross sectional studies: adapted form of NOS Low (scored 8–9), Medium (scored 6–7), High risk of bias (scored ≤5).	Obstructive Sleep Apnoea: Overnight polysomnography conducted at a sleep laboratory (type I study) or at home using a portable monitor (type II study). Types III and IV home-based sleep testing. Epworth Sleepiness Scale [ESS], Berlin Questionnaire, Stanford Sleepiness Scale, STOP or STOP BANG, Apnea Risk Evaluation System [ARES])	Periodontitis: Primary outcome measures: Clinical Attachment Loss (CAL), Periodontal Probing Depth (PPD), radiographically assessed Alveolar Bone Loss (ABL), oral hygiene indices. Secondary outcome measures: Levels of salivary inflammatory mediators (cytokines including tumor necrosis factoralpha [TNF-α], interleukins, or levels of the enzyme aspartate aminotransferase in the gingival crevicular Fluid	Meta-analysis	Not assessed	There is some evidence to a plausible association between periodontal disease and OSA
Schmidlin 2020 (20)	Zurich	2020	5 Databases: MEDLINE, PsycINFO, Cochrane library, Web of Science, Scopus	February 3 2020 to February 11, 2020	13 studies: 9 cross-sectional, 3 case-control 1 retrospective cohort study	200,145	Newcastle Ottawa Scale (NOS): Low risk of bias (≥7 stars), Moderate risk of bias (5–6 stars), High risk of bias (≤4 stars).	Non-apnea sleep disorder Pittsburg Sleep Quality Index(PSQI) Epworth Sleepiness Scale(ESS) Self-reported Average of Daily Sleep duration(SADS)	Periodontal disease Clinical Attachment Loss(CAL) Radiographic Attachment Loss(RAL) Pocket Probing Depth(PPD) Papillary Bleeding Index(PBI) Plaque Index(PI)–Modified Gingival Index Approximal Plaque Index(API) Community Periodontal Index(CPI) Community Periodontal Index of Treatment Needs(CPITN)	Narrative synthesis	Not assessed	Sleep initiation and maintenance disorders have been found repeatedly to be associated with periodontitis
Liu 2022 (38)	China	2022	3 Databases: PubMed, Web of Science, Embase	From inception to March 26 2022	10 studies: 2 case-control, 8 cross sectional	43,296	Newcastle Ottawa Scale (NOS):1 to 9	Sleep-disordered breathing: Apnoea-Hypopnea Index(AHI): Mild: AHI ≥5 to <15 events per hour, Moderate: AHI ≥ 15 to <30 events per hour, Severe: AHI ≥30 events per hour	Periodontitis: American Academy of Periodontology(AAP) or Centre for Disease Control and Prevention(CDC) definitions: Mild periodontitis: ≥2 interdental sites with clinical attachment level (CAL) ≥3 mm and ≥2 interdental sites with probing depth (PD) ≥4 mm (not in the same tooth), or one site with PD ≥ 5 mm. Moderate periodontitis: ≥2 interdental sites with CAL ≥4 mm (not in the same tooth), or ≥2 interdental sites with PD ≥ 5 mm.—Severe Periodontitis: ≥2 interdental sites with CAL ≥6 mm (not in the same tooth) and ≥1 interdental site with PD ≥ 5 mm	Meta-analysis	Assessed	Polysomography confirmed diagnosis of sleep disordered breathing is associated with periodontitis among adults
Muniz 2021 (13)	Brazil	2021	3 Databases: PubMed, Scopus, and Embase	Between January 1952 and May 2021	20 studies: 16 cross-sectional, 2 case-control, 2 cohorts	232,403	Cross-sectional studies: Agency for Healthcare Research and Quality (AHRQ) scale Case control and Cohort studies: Newcastle-Ottawa quality assessment scale	Sleep duration or Sleep quality: Pittsburg Sleep Quality Index(PSQI)	Periodontal disease: Community Periodontal Index (CPI) Clinical Attachment Loss(CAL) Probing Depth(PD)	Narrative synthesis	Not Assessed	Conflicting results for the association between sleep hours and periodontitis. However, inadequate sleep may be associated with lower number of present teeth and periodontal diseases
Molina 2022 (39)	Spain	2022	3 Databases: PubMed, Cochrane Library (including Cochrane Database for Systematic Reviews and Cochrane CENTRAL register for Clinical Trials); Scopus	Upto October 2021	12 studies: 7 Cross-sectional, 5 case-control	47,024	Cross-sectional studies: Joanna Bridge Institute(JBI) critical appraisal tool Case- control studies: Modification of the Newcastle Ottawa Scale (NOS)	Obstructive sleep apnoea: Polysomnography Berlin's questionnaire STOP Questionnaire STOP-BANG Questionnaire Apnoea-Hypopnea Index(AHI)	Periodontitis: American Dental Association/Academy of Periodontology/Centre for Disease Control and Prevention criteria, Plaque Index(PD) Gingival Index(GI) Calculus Index Probing Depth(PD) Clinical Attachment Loss(CAL) Bleeding on Probing(BoP) Gingival Recession(REC)	Meta-analysis	Assessed	Supports an association between periodontitis and OSA
Feixiang Zhou 2021 (37)	China	2021	3 Databases: PubMed, Web of Science EMBASE	Upto 23 September 2020	7 Cross-sectional studies,	40,196	Agency for Healthcare Research and Quality (AHRQ) with scores ranging from 0 to 11 points >8 points: high quality.	Sleep duration—Self-reported hours of sleep	Periodontitis: Clinical Attachment Loss(CAL)—Modified Gingival Index(GI)—Community Periodontal Index(CPI)	Meta-analysis	Assessed	Short sleep is not associated with the risk of periodontal disease

The systematic reviews included case-control, cohort, and cross-sectional study designs. Among these, cross-sectional studies were utilized across all reviews. The Newcastle-Ottawa Scale was the predominant tool used to assess study quality (13, 15–17, 19, 20, 34, 35, 38, 39). Other quality assessment methods employed included the Agency for Healthcare Research and Quality (AHRQ) (18, 36, 37) and JBI critical appraisal tool (39).

Regarding exposure parameters, the systematic reviews evaluated various aspects of sleep, including:

- Sleep Duration: Measured in hours.
- Sleep Disorders: Such as obstructive sleep apnea (OSA) and sleep-disordered breathing.
- Non-Apnea Sleep Disorders: Other sleep-related disorders not classified as apnea.

Methods used to assess the sleep across these reviews include polysomnography (15–17, 35, 39), Apnoea- Hypopnea Index (15, 19, 34, 38, 39), Pittsburgh sleep Quality Index (13, 20), sleep questionnaires (16, 17, 35, 36, 39) etc. The method of outcome assessment varied among the reviews, with diagnostic parameters commonly used being clinical attachment loss and bleeding on probing. Meta-analysis was utilized in seven reviews to synthesize study findings (16–18, 36–39), while the remaining six reviews employed narrative synthesis (13, 15, 19, 20, 34, 35).Publication bias was evaluated in six reviews (17, 18, 36–39). One review established an association between sleep and the occurrence of periodontal disease (19). Four reviews examined the relationship between short sleep and periodontal disease (13, 36–38), with 3 reviews identifying a positive association (13, 36, 38). Six reviews investigated the association between sleep disorders, with a focus on obstructive sleep apnea (OSA), and periodontal disease (15–17, 34, 35, 39): Two reviews found a positive association between OSA and periodontal disease (17, 34). Out of which one review suggested that obstructive sleep apnea and related conditions might influence the composition of oral microbiota (34). One review indicated that periodontitis is associated with mild-to-moderate OSA but not with severe OSA (17). Two reviews provided plausible explanations for the link between OSA and periodontal disease (16, 35). One review found limited evidence of an association between OSA and periodontal disease (15). One review explored the association between sleep-disordered breathing and periodontal disease, reporting a positive association (38). One review assessed the link between non-apnea sleep disorders and periodontal disease, also reporting a positive association (20). Out of the 13 reviews, seven included meta-analyses (16–18, 36–39). Among these Five reviews identified a positive association between sleep and periodontitis (16–18, 38, 39). Two reviews identified a negative association (36, 37).

### 3.3 Methodological quality

Among the 11 quality appraisal criteria outlined in the JBI Critical Appraisal Instrument for Systematic Reviews and Research Syntheses, three of the included systematic reviews met 10 criteria each (18, 37, 39) (Table 2). However, the remaining ten reviews did not meet some criteria or were rated as unclear due to insufficient reporting (13, 15–17, 19, 20, 34–36, 38). In six systematic reviews, the review question was not explicitly stated could be inferred from the title or abstract (13, 17, 19, 36–38). It was noted in the assessment that two systematic reviews did not clearly specify their inclusion criteria (34, 36), and one systematic review failed to specify them entirely (19). The search strategy was inadequately framed in eight systematic reviews (13, 15, 17–20, 35, 36). Additionally in two systematic reviews, the databases used for the search strategy were deemed inadequate (19, 35). Moreover, the criteria used for study appraisal were not mentioned in six systematic reviews (15–17, 19, 20, 38) and were unclear in one review (13). Furthermore, one systematic review did not clearly specify whether critical appraisal were conducted independently by two or more reviewers (19). All systematic reviews adopted methods to minimize errors in data extraction. However, methods used to combine studies were unclear in two reviews (13, 34), and were not mentioned in two others (15, 19). Publication bias was assessed in 6 systematic reviews (17, 18, 36–39). Seven reviews included recommendations for policy and practice, indicating practical implications of their findings (13, 17, 18, 34–37) With one exception (34), all reviews also proposed specific directives for new research highlighting areas where future reviews could build upon their findings.

**Table 2:** Appraisal of methodological quality of the included systematic reviews.

JBI Critical Appraisal items	1	2	3	4	5	6	7	8	9	10	11
Lembo 2021	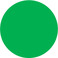	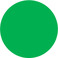	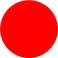	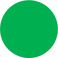	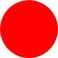	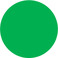	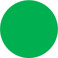	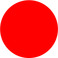	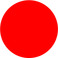	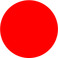	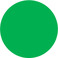
Wu 2022	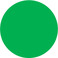	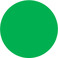	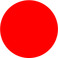	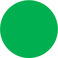	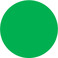	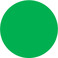	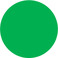	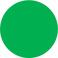	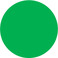	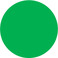	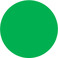
Qun Zhou 2024	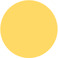	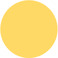	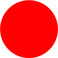	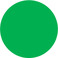	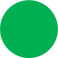	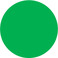	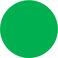	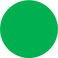	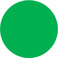	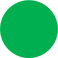	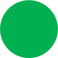
Edoardo Bianchi 2022	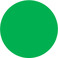	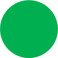	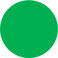	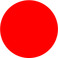	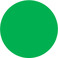	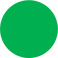	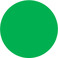	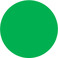	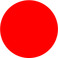	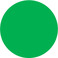	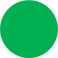
Giorgio Bianchi 2022	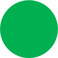	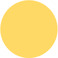	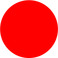	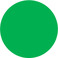	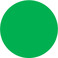	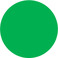	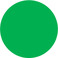	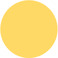	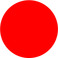	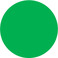	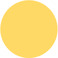
Khodadadi 2022	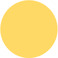	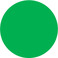	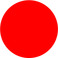	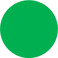	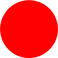	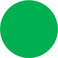	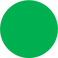	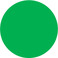	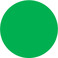	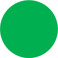	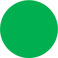
Malaiappan 2019	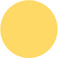	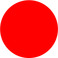	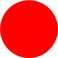	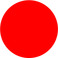	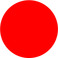	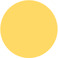	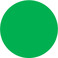	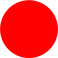	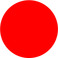	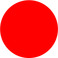	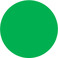
Al-Jewair 2015	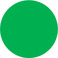	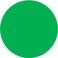	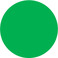	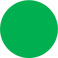	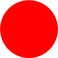	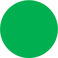	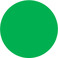	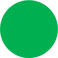	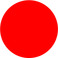	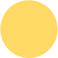	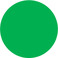
Schmidilin 2020	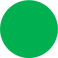	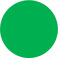	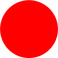	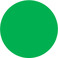	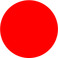	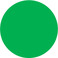	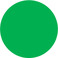	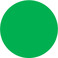	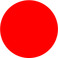	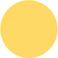	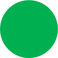
Liu 2022	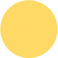	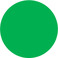	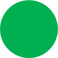	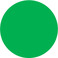	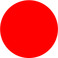	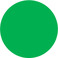	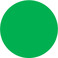	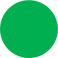	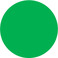	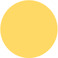	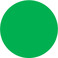
Muniz 2021	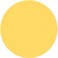	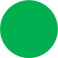	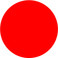	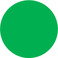	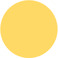	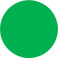	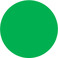	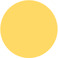	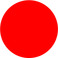	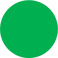	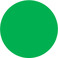
Molina 2022	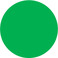	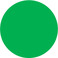	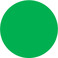	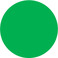	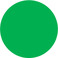	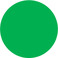	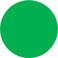	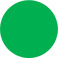	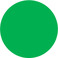	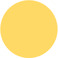	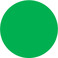
Feixiang Zhou 2021	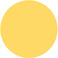	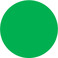	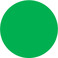	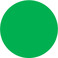	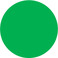	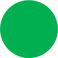	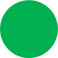	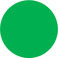	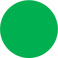	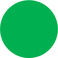	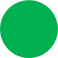

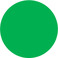
 = Yes. 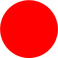 = No. 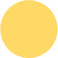 = Unclear, NA, not applicable.

Scoring: Yes = 3, Unclear = 2, No = 1, Not applicable=0.

1: Is the review question clearly and explicitly stated? 2: Were the inclusion criteria appropriate for the review question? 3: Was the search strategy appropriate? 4: Were the sources and resources used to search for studies adequate? 5: Were the criteria for appraising studies appropriate? 6: Was critical appraisal conducted by two or more reviewers independently? 7: Were there methods to minimize errors in data extraction? 8: Were the methods used to combine studies appropriate? 9: Was the likelihood of publication bias assessed? 10: Were recommendations for policy and/or practice supported by the reported data? 11: Were the specific directives for new research appropriate?

Due to the limited number of trials and small sample sizes, the GRADE evaluation of combined outcomes in this umbrella review resulted in trials being downgraded for impression and risk of bias. Consequently, more caution was exercised in the overall interpretation of the data. Overall, 11 reviews were rated as good quality, indicating low risk of bias, while two reviews were rated as moderate quality, indicating higher risk of bias.

### 3.4 Review of findings

This umbrella review synthesizes findings from seven systematic reviews investigating the relationship between sleep and occurrence of periodontal disease through meta- analysis Wu synthesized data from 107,777 participants across 6 studies, reporting an odds ratio of 1.19 (95% CI 1.16–1.23), and no heterogeneity(I^2^ = 0%) (18). Zhou assessed the association of both short and long sleep duration with occurrence of periodontal disease (36). Zhou analyzed short sleep duration with occurrence of periodontal disease, across 10 studies involving 66,516 participants, finding an odds ratio of 1.04 (95% CI 0.83–1.29) with high heterogeneity (*I*^2^ = 94.4%). Conversely Zhou evaluated long sleep duration and occurrence of periodontal disease, across 8 studies involving 64,449 participants, reporting an odds ratio of 1.12 (95% CI 0.94–1.33), with low heterogeneity (*I*^2^ = 38.6%). Khodadadi (2022) reviewed 10 studies comprising 30,994 participants, revealing a stronger association with an odds ratio of 2.17 (95%CI 1.66–2.83) and moderate heterogeneity (*I*^2^ = 43%) (17). Al- Jewair (2015) assessed 4 studies comprising 29,995 participants reporting an odds ratio of 1.65 (95% CI 1.11–2.46) with high heterogeneity (*I*^2^ = 92%) (16). Liu (2022) analyzed 10 studies with 43,296 participants, observing an odds ratio of 1.83(95% CI 1.52–2.20) with low heterogeneity (*I*^2^ = 40%) (38). Molina (2022) included 6 studies with 43,342 participants, finding an odds ratio of 1.65 (95% CI 1.21–2.25) with substantial heterogeneity (*I*^2^ = 86.5%) (39). Feixiang Zhou (2024) evaluated 7 studies with 40,196 participants, reporting an odds ratio of 1.06 (95% CI 0.89–1.25) with substantial heterogeneity (*I*^2^ = 72.8%) (37).

A simple visual indication of the results followed a stoplight pattern to reflect the presence or absence of an association between exposure and outcome (Table 3) A green coloured stoplight denoted a positive association between sleep and periodontitis, as reported in five of the included reviews (16–18, 38, 39), while a red- coloured stoplight denoted a negative association, as reported in two reviews (36, 37).

**Table 3:** Traffic stop light.

Systematic review	Number of studies	Number of participants	Odd's ratio	Upper limit	Lower limit	GRADE	Heterogeneity	Stoplight
Wu 2022	6	1,07,777	1.19	1.16	1.23	Not assessed	0%	
Zhou 2024a -Short sleep duration	10	66,516	1.04	0.83	1.29	Not assessed	94.4%	
Zhou 2024b - Long sleep duration	8	64,449	1.12	0.94	1.33	Not assessed	38.6%	
Khodadadi 2022	10	30,994	2.17	1.66	2.83	Not assessed	43%	
Al-Jewair 2015	4	29,955	1.65	1.11	2.46	Not assessed	92%	
Liu 2022	10	43,296	1.83	1.52	2.2	Not assessed	40%	
Molina 2022	6	43,342	1.65	1.21	2.25	Not assessed	86.5%	
Feixiang Zhou 2021	7	40,196	1.06	0.89	1.25	Not assessed	72.8%	

Association between sleep and periodontal disease □.

No Association between sleep and periodontal disease □.

**Question:** What is the association of sleep with occurrence of periodontal disease?.

Population: Adults >18 years of age.

Exposure: Sleep.

Outcome: Periodontal Disease.

Overall, these reviews consistently suggest a positive association between poor sleep and the incidence of periodontal disease as indicated by odds ratio greater than 1. However, the effect sizes vary, ranging from modest (OR 1.04–1.19) to more pronounced (OR 1.65–2.17), indicating differing strengths of association across the reviews. Heterogeneity among included reviews is notable, with I² values ranging from 0% to 94.4%, suggesting variability in methodologies, populations, or other factors influencing the outcomes.

### 3.5 Summary of findings of Umbrella review for certainty assessment

The eight cross-sectional studies included in the umbrella review assessed the relationship between adult sleep disorders and periodontal disease. (Table 4). Using the GRADE framework, the level of evidence certainty was determined to be low. Several factors are reflected in this rating: Because of the limitations of cross-sectional designs, the studies showed a very serious risk of bias, which calls into question the validity of the results. Despite the high levels of heterogeneity in the reviews, this was not regarded as a significant problem for consistency. Because there was an appropriate number of studies and participants, the evidence was directly relevant to the research question, with no significant indirectness, and it did not show serious imprecision. Although there was no strong evidence of publication bias, there is a chance that the effects that were seen were spurious due to plausible residual confounding. As a result, the low certainty rating suggests that more investigation using stricter methodologies is needed to gain a deeper understanding of the connection between periodontal disease and breathing disorders during sleep.

**Table 4:** Summary of findings of Umbrella review for certainty assessment.

Certainty assessment								Certainty
№ of studies	Study design	Risk of bias (Quality of Included reviews)	Inconsistency presence of heterogeneity in included reviews	Indirectness direct association	Imprecision number of studies in each review and number of participants in review	Other considerations publication bias low effect size in each review plausible confounding adjustments in reviews	Relative (95% CI)	
8	Cross sectional studies in the included review	Extremely serious	Not serious	Not serious	Not serious	All plausible residual confounding would suggest spurious effect, while no effect was observed	Not estimable	⨁⨁ˆˆ Low

**Question:** What is the association of sleep with occurrence of periodontal disease?.

Population: Adults.

Exposure: Sleep, Obstructive Sleep Aponea.

Outcome: Occurrence of Periodontal Disease.

Explanations.

a. All included reviews are based on PEO, including cross-sectional or case-control designs. Heterogeneity is high in all reviews.

b. The GRADE framework includes evaluation of the following five criteria.

c. i) quality of primary studies (e.g., risk of bias and methodological limitations).

d. ii) inconsistency [e.g., direction of intervention effects, magnitude of statistical heterogeneity measured by *I*^2^; low (*I*^2^, 0- 40%), moderate (*I*^2^ 40–60%), high (I^2^ > 60%).

e. Iii) indirectness (e.g., direct comparisons with populations, interventions and outcomes relevant to context).

f. iv) imprecision (e.g., magnitude of the number of included studies: large: >10 studies, moderate: 5–10 studies, small: <5 studies and median sample size; high >300 participants, intermediate 100–300 participants, low <100 participants) and.

g. v) publication bias

### 3.6 Assessment of publication overlap and corrected covered area (CCA) index

**Figure 2:**
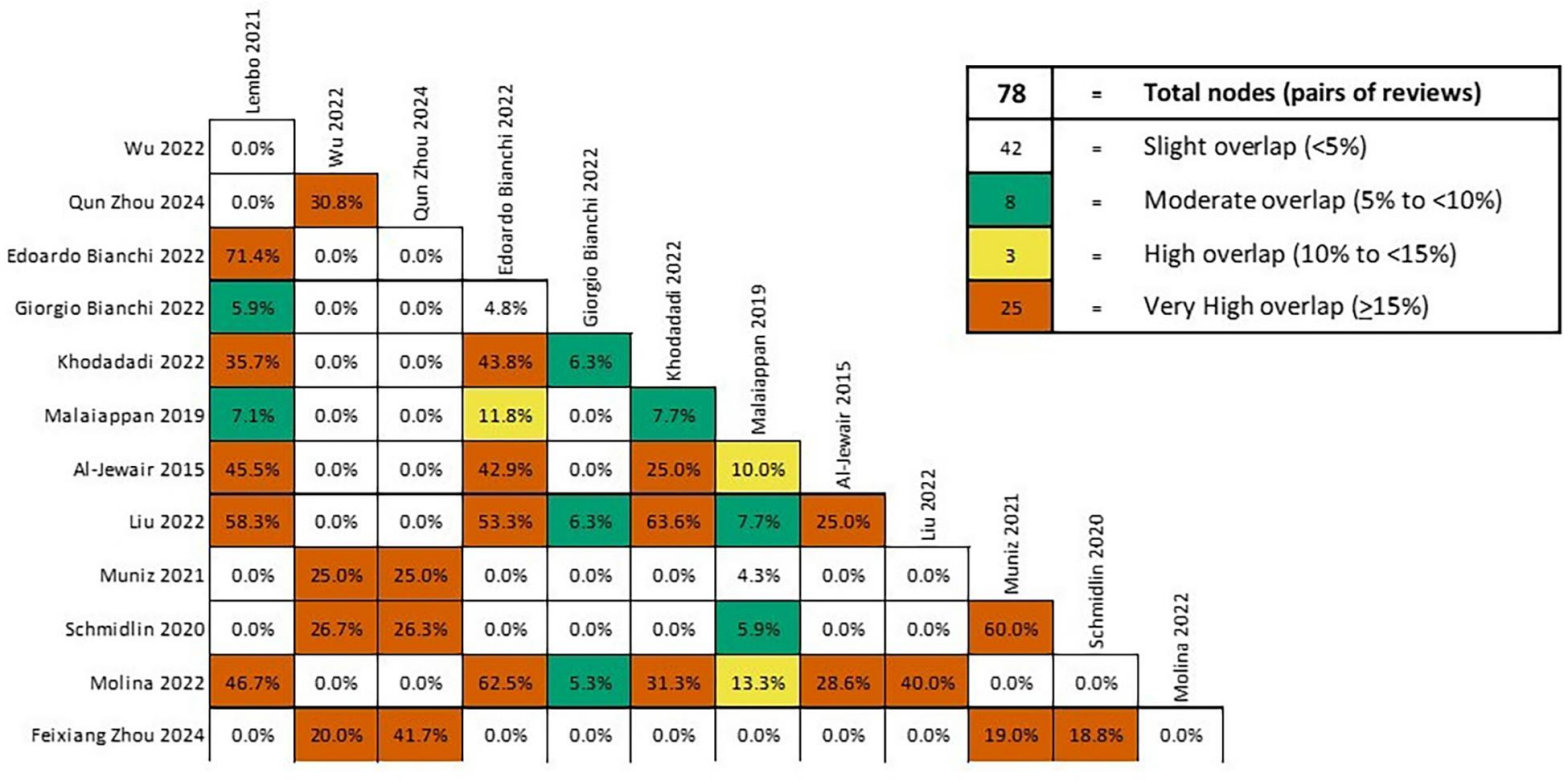
Overlap of primary studies in included reviews.

The overlap assessment of the systematic reviews in this umbrella review highlights a very high degree of overlap, with an overall covered area of 15%. The corrected covered area (CCA) was calculated as 12.18%, indicating minimal duplication. This overlap distribution suggests that, the systematic reviews include repeated primary studies, potentially impacting the synthesis of results. Besides calculating an overall CCA for the whole matrix, calculation of CCA for every “node” (or possible pair of systematic reviews within the matrix)- the results were visually summarized in Figure 2 in the form of Heat map generated using the GROOVE tool. There were 78 total nodes (pair of reviews), of which 42 had slight overlap (<5%), 8 had moderate overlap (5% to <10%), 3 had high overlap (10% to <15%) and 25 had very high overlap (>15%).

## 4 Discussion

While periodontal disease is globally recognized as a significant public health concern (40), sleep remains under-recognized, under-reported, and insufficiently prioritized (41). According to Lancet May 2024 reports, sleep should no longer be neglected as a public health issue (42), as it is a fundamental daily activity that, when disrupted, can have profound implications for systemic health, thereby exacerbating the existing burden of diseases. Sleep may play a crucial role in the development of periodontal disease, as both conditions are closely linked to inflammation. Sleep disturbances can alter biomarker levels, such as C-reactive protein (CRP), tumor necrosis factor-alpha (TNF-α), and interleukin-6 (IL-6), all of which are implicated in the inflammatory processes underlying periodontal disease (13, 43). Inadequate sleep can negatively impact cognitive function and motor skills (44), which may hinder a person's ability to perform proper oral hygiene practices and, consequently, increase the risk of periodontal disease (45). Given these interconnections, it is essential to carefully examine the potential association between sleep and periodontal disease, and to understand how this synergism might affect quality of life. By doing so, we can develop strategies to prevent further complications arising from the interaction between these two conditions.

Compared with prior systematic reviews—such as Al-Jewair et al., which focused on obstructive sleep apnoea, and the more recent meta-analyses by Khodadadi and Liu, which reported larger pooled effect estimates—this umbrella review goes beyond simple aggregation. It synthesizes existing syntheses, quantifies the degree of primary-study overlap (CCA = 12.18%), and applies the GRADE approach to provide a structured assessment of certainty. Accordingly, the principal contribution of this work is methodological: it demonstrates that much of the apparent consistency in the literature arises from repeated inclusion of the same primary studies, and it highlights where truly independent replication remains limited.

### 4.1 Sleep duration and periodontal disease

The review indicates that alterations in sleep duration are associated with periodontal disease (19). This finding highlights the importance of maintaining adequate sleep for periodontal health, though further research is needed to elucidate the specific mechanisms driving this relationship.

### 4.2 Short sleep and periodontal disease

The available evidence suggests that short sleep may be a significant factor in the development or progression of periodontal disease, as supported by consistent positive associations in several reviews (13, 18, 36, 37). However, further studies are necessary to clarify the underlying mechanisms.

### 4.3 Sleep disorders and periodontal disease

The association between sleep disorders, especially obstructive sleep apnea (OSA), and periodontal disease presents several key findings: The positive associations identified in some reviews (34, 39) along with plausible explanations provided in others (16, 35), suggest that OSA and related conditions might influence periodontal health, primarily through mechanisms such as changes in oral microbiota (34). The observed link between mild-to-moderate, but not severe, OSA and periodontal disease implies a threshold effect, where only certain levels of OSA severity are associated with periodontal issues (17). More detailed research is required to address reviews reporting limited evidence, to achieve a more precise understanding (15).

### 4.4 Sleep-Disordered breathing and non-apnea sleep disorders

The positive association between sleep-disordered breathing and periodontal disease, as well as the link with non-apnea sleep disorders reported in some reviews (20, 38) indicates that various types of sleep disturbances can impact periodontal health. This suggests that a broader view of sleep-related issues should be considered when assessing their effects on periodontal disease.

The meta-analyses concludes that poor sleep is generally associated with periodontitis, with five of the seven reviews reporting a positive association. However, the contradictory findings in the remaining two reviews highlight the need for more strong and consistent evidence to fully understand the relationship between sleep disturbances and periodontal disease. Further research should aim to address these gaps and explore the factors contributing to the observed variations in outcomes.

The negative associations obtained from the reviews were attributed to several factors. These include heterogeneity in the classification of periodontal disease, variability in the methods used to assess the severity of obstructive sleep apnea (OSA) (15) and measure sleep duration, recall bias, and the exclusion of certain subtypes of periodontal disease, such as gingivitis and tooth mobility. All these could be some of the potential factors which distorts the estimate of the true association. Although all reviews have undoubtedly stated the role of inflammatory mediators in the pathogenesis of both sleep disorders and periodontitis, the causal-effect relationship of periodontal disease was debatable in most of the reviews due to the conflicting results.

The strength of association between poor sleep and periodontal disease varies across the included reviews. Reviews such as Khodadadi 2022 and Liu 2022 consistently demonstrate a strong positive association, with odds ratios of 2.17 (95% CI: 1.66–2.83) and 1.83 (95% CI: 1.52–2.20) respectively, indicating a significant increase in the likelihood of periodontal disease among those with poor sleep. In contrast, reviews like Wu 2022 and Zhou 2024b, focusing on long sleep duration, show weaker associations with odds ratios around 1.19 (95% CI: 1.16–1.23) and 1.12 (95% CI: 0.94–1.33) respectively. Furthermore, reviews examining short sleep duration, such as Zhou 2024a, and those exploring general sleep patterns, like Feixiang Zhou 2021, do not demonstrate statistically significant associations. Some reviews included in the meta-analysis had a high level of confidence which had strengthened the evidence for results. However more rigorous research should be conducted in reviews with lower confidence levels. Overall, the combined strength of association across these reviews suggests a consistent trend towards an increased risk of periodontal disease with poor sleep, particularly in reviews with higher odds ratios and narrower confidence intervals.

This umbrella review synthesizes evidence from multiple systematic reviews to provide a structured and contextual overview of the association between sleep and periodontal disease. Beyond integrating existing findings, it adds to the literature by systematically comparing prior syntheses, quantifying overlap of primary studies using the corrected covered area (CCA), and assessing the certainty of evidence through the GRADE approach—methodological elements that were inconsistently applied or absent in earlier reviews. The use of comprehensive database searches, independent study selection and data extraction, and standardized quality appraisal enhances the transparency and reliability of the synthesis. In addition, the inclusion of reviews encompassing diverse populations allows broader contextualization of findings. Collectively, these strengths support a coherent interpretation of the current evidence base while acknowledging its methodological variability and limitation.

Despite the valuable insights provided by this umbrella review, several important limitations warrant consideration. Although the literature search was broad and recognized appraisal frameworks [JBI methodology, GRADE, and corrected covered area (CCA)] were applied, the synthesized evidence was characterized by substantial heterogeneity, extensive overlap of primary studies, and overall low certainty of evidence, which collectively weaken the strength of the conclusions. A key challenge lies in the lack of consistency in the definition and measurement of both sleep exposures and periodontal disease outcomes. Sleep was variably assessed as sleep duration, subjective sleep quality, or specific sleep disorders such as obstructive sleep apnea, using heterogeneous questionnaire-based and objective measures, while periodontal disease was evaluated using different clinical indices, case definitions, and, in some instances, self-reported outcomes. These variations limit comparability across studies and likely contribute to inconsistent findings.

In addition, the assessment of multiple sleep dimensions increases the risk of spurious associations, particularly given inconsistent adjustment for important confounders such as age, lifestyle factors, and systemic health conditions. Although disease severity was considered in some reviews, ongoing periodontal treatment status was not uniformly addressed, potentially influencing outcome estimates. Publication and recall biases, along with variable methodological quality of the included reviews, further reduce confidence in the synthesized findings. Finally, the predominance of cross-sectional evidence and the limited availability of prospective and interventional studies restrict inference regarding temporality and causality. Taken together, these limitations necessitate cautious interpretation of the findings and highlight the need for methodologically robust future research.

### 4.5 Implications for clinical practice

Oral health professionals are encouraged to raise awareness among patients regarding the association between poor sleep and the development of periodontal disease, and to offer guidance on optimal sleep hygiene practices. Screening tools should be developed for dental settings to assess sleep, aiding in the identification of individuals at risk for periodontal disease. Comprehensive case-history taking during clinical evaluations should encompass potential sleep-disrupting factors such as anxiety, stress, depression, snoring, obstructive sleep apnea, work mode and duration, and smoking status. The implementation of AI-powered wearable devices and mobile applications for evaluating sleep is advised. A holistic approach involving collaboration among sleep specialists, neurologists, psychologists, dental professionals, and public health professionals is essential to ensure coordinated medical and dental care delivery.

### 4.6 Implications of future research

Future studies should clarify whether the relationship between sleep and periodontal disease is bi-directional and how variations in sleep duration—short or long—affect periodontal outcomes. Research is needed to determine whether improving sleep leads to better periodontal health. Prospective studies focusing on sleep duration and quality, rather than only sleep disorders, are essential to establish temporal and causal links. Standardised periodontal diagnostic criteria will improve comparability across studies. Special attention should be given to high-risk groups such as shift workers, individuals with systemic diseases, people with disabilities, and those experiencing psychological distress, who may be more vulnerable to both sleep disturbances and periodontal disease.

## 5 Conclusion

Low-certainty evidence from multiple systematic reviews indicates a possible association between sleep disturbances (including OSA and short sleep duration) and periodontal disease in adults. However, due to high heterogeneity, extensive overlap of primary studies, predominance of cross-sectional designs, and inconsistent exposure/outcome definitions, causality cannot be established. High-quality prospective studies using standardised sleep and periodontal measures are required.

## Data Availability

The datasets presented in this study can be found in online repositories. The names of the repository/repositories and accession number(s) can be found in the article/Supplementary Material.
