# Supplementary material for: Association between sleep and periodontal disease in adults—an umbrella review
Source: Front Oral Health. 2026 Mar 12;7:1761243. doi: 10.3389/froh.2026.1761243 (PMC13018118; doi:10.3389/froh.2026.1761243)
Supplement: Supplementary file 1 [file Table1.docx]

**Supplementary file 1 : Search strategy**

**Medline Ovid**

Search date and time : 21/3/2024, 1:30 pm: Retrieved 7 results

| Sl.No | Query | Records retrieved |
| --- | --- | --- |
| 1 | exp Young Adult/ or exp Adult Children/ or exp Adult/ or adult.mp. | 8560082 |
| 2 | aged.mp. or Aged/ | 6032337 |
| 3 | 1 or 2 | 8877312 |
| 4 | "sleep disorders".mp. or exp Sleep Wake Disorders/ | 119362 |
| 5 | "sleep deprivation".mp. or exp Sleep Deprivation/ | 15278 |
| 6 | "sleep arousal disorders".mp. or Sleep Wake Disorders/ or exp Sleep Arousal Disorders/ or exp Sleep Apnea, Obstructive/ | 56237 |
| 7 | "sleep duration".mp. or exp Sleep Duration/ | 13481 |
| 8 | 4 or 5 or 6 or 7 | 130178 |
| 9 | periodontitis.mp. or exp Periapical Periodontitis/ or exp Chronic Periodontitis/ or exp Aggressive Periodontitis/ or exp Periodontitis/ | 50363 |
| 10 | exp Periodontitis/ or periodont*.mp. or exp Periodontal Diseases/ | 144437 |
| 11 | 9 or 10 | 144437 |
| 12 | 3 and 8 and 11 | 107 |
| 13 | limit 12 to "review articles" | 7 |

**Scopus**

Search date and time : 25/3/2024, 10:36 am: Retrieved 10 results

| Sl.No | Query | Records retrieved |
| --- | --- | --- |
| #1 | ( TITLE-ABS-KEY ( adult ) OR TITLE-ABS-KEY ( "young adult" ) OR TITLE-ABS-KEY ( "adult children" ) OR TITLE-ABS-KEY ( aged ) ) | 11,823,879 |
| #2 | ( TITLE-ABS-KEY ( "sleep disorders" ) OR TITLE-ABS-KEY ( "sleep deprivation" ) OR TITLE-ABS-KEY ( "sleep disorders, intrinsic" ) OR TITLE-ABS-KEY ( "sleep arousal disorders" ) OR TITLE-ABS-KEY ( "sleep apnoea, obstructive" ) OR TITLE-ABS-KEY ( "sleep wake disorders" ) OR TITLE-ABS-KEY ( "sleep duration" ) ) | 152308 |
| #3 | ( TITLE-ABS-KEY ( periodontitis ) OR TITLE-ABS-KEY ( "chronic periodontitis" ) OR TITLE-ABS-KEY ( "aggressive periodontitis" ) OR TITLE-ABS-KEY ( periodont* ) ) | 151048 |
| #4 | ( ( TITLE-ABS-KEY ( adult ) OR TITLE-ABS-KEY ( "young adult" ) OR TITLE-ABS-KEY ( "adult children" ) OR TITLE-ABS-KEY ( aged ) ) ) AND ( ( TITLE-ABS-KEY ( "sleep disorders" ) OR TITLE-ABS-KEY ( "sleep deprivation" ) OR TITLE-ABS-KEY ( "sleep disorders, intrinsic" ) OR TITLE-ABS-KEY ( "sleep arousal disorders" ) OR TITLE-ABS-KEY ( "sleep apnoea, obstructive" ) OR TITLE-ABS-KEY ( "sleep wake disorders" ) OR TITLE-ABS-KEY ( "sleep duration" ) ) ) AND ( ( TITLE-ABS-KEY ( periodontitis ) OR TITLE-ABS-KEY ( "chronic periodontitis" ) OR TITLE-ABS-KEY ( "aggressive periodontitis" ) OR TITLE-ABS-KEY ( periodont* ) ) ) AND ( LIMIT-TO ( DOCTYPE , "re" ) ) | 10 |

**Cinahl**

Search date and time: 25/3/24, 1:06 pm: Retrieved 127 results

| Sl.No | Query | Records retrieved |
| --- | --- | --- |
| S1 | adult OR young adults OR adult children OR aged | 12,823,170 |
| S2 | sleep disorders OR sleep deprivation OR sleep disorders, intrinsic OR sleep arousal disorders OR sleep apnea, obstructive OR sleep wake disorders OR sleep duration | 272,132 |
| S3 | periodontitis OR chronic periodontitis OR aggressive periodontitis OR periodont* | 306,765 |
| S4 | ( adult OR young adults OR adult children OR aged ) AND ( sleep disorders OR sleep deprivation OR sleep disorders, intrinsic OR sleep arousal disorders OR sleep apnea, obstructive OR sleep wake disorders OR sleep duration ) AND ( periodontitis OR chronic periodontitis OR aggressive periodontitis OR periodont* ) | 127 |

**Cochrane**

Search date and time: 22/3/24 11:44 am: Retrieved 2 results

| S.No | Query | Records retrieved |
| --- | --- | --- |
| 1 | adult | 803,884 |
| 2 | MeSH descriptor: [Adult] explode all trees | 612,808 |
| 3 | "young adult" | 111,924 |
| 4 | MeSH descriptor: [Young Adult] explode all trees | 95,267 |
| 5 | "adult children" | 15,207 |
| 6 | MeSH descriptor: [Adult Children] explode all trees | 37 |
| 7 | aged | 648,523 |
| 8 | MeSH descriptor: [Aged] explode all trees | 273,978 |
| 9 | #1 OR #2 OR #3 OR #4 OR #5 OR #6 OR #7 OR #8 | 1,044,831 |
| 10 | "sleep disorders" | 3,363 |
| 11 | MeSH descriptor: [Sleep Wake Disorders] explode all trees | 12,247 |
| 12 | "sleep deprivation" | 2,121 |
| 13 | MeSH descriptor: [Sleep Deprivation] explode all trees | 1,105 |
| 14 | "sleep disorders, intrinsic" | 24 |
| 15 | MeSH descriptor: [Sleep Disorders, Intrinsic] explode all trees | 8,611 |
| 16 | "sleep arousal disorders" | 7 |
| 17 | MeSH descriptor: [Sleep Arousal Disorders] explode all trees | 20 |
| 18 | "sleep apnea, obstructive" | 3,186 |
| 19 | MeSH descriptor: [Sleep Apnea, Obstructive] explode all trees | 3,181 |
| 20 | "sleep wake disorders" | 2,409 |
| 21 | MeSH descriptor: [Sleep Wake Disorders] explode all trees | 12,247 |
| 22 | "sleep duration" | 2,189 |
| 23 | MeSH descriptor: [Sleep Duration] explode all trees | 18 |
| 24 | #10 OR #11 OR #12 OR #13 OR #14 OR #15 OR #16 OR #17 OR #18 OR #19 OR #20 OR #21 OR #22 OR #23 | 16,878 |
| 25 | periodontitis | 7,251 |
| 26 | MeSH descriptor: [Periodontitis] explode all trees | 3,921 |
| 27 | "chronic periodontitis" | 3,125 |
| 28 | MeSH descriptor: [Chronic Periodontitis] explode all trees | 1,112 |
| 29 | "aggressive periodontitis" | 290 |
| 30 | MeSH descriptor: [Aggressive Periodontitis] explode all trees | 139 |
| 31 | periodont* | 17,976 |
| 32 | MeSH descriptor: [Periodontitis] explode all trees | 3,921 |
| 33 | #25 OR #26 OR #27 OR #28 OR #29 OR #30 OR #31 OR #32 | 18,014 |
| 34 | #9 AND #24 AND #33 in Cochrane Reviews | 2 |

**Proquest**

Search date and time: 25/3/2024, 11:12 am: Retrieved 12 results

| S.No | Query | Records retrieved |
| --- | --- | --- |
| S1 | adult OR "young adult" OR "adult children" OR aged | 1787864 |
| S2 | "sleep disorders" OR "sleep deprivation" OR "sleep disorders, intrinsic" OR "sleep arousal disorders" OR "sleep apnoea, obstructive" OR "sleep wake disorders" OR "sleep duration" | 51609 |
| S3 | periodontitis OR "chronic periodontitis" OR "aggressive periodontitis" OR periodont* | 29597 |
| S5 | ([S1] AND [S2] AND [S3]) AND at.exact("Literature Review" OR "Review") | 12 |
|  |  |  |
|  |  |  |
|  |  |  |
|  |  |  |
|  |  |  |
|  |  |  |
|  |  |  |
|  |  |  |
|  |  |  |
|  |  |  |
|  |  |  |
|  |  |  |
|  |  |  |
|  |  |  |
|  |  |  |
|  |  |  |
|  |  |  |
|  |  |  |
|  |  |  |
|  |  |  |
|  |  |  |
|  |  |  |
|  |  |  |
|  |  |  |
|  |  |  |
|  |  |  |
|  |  |  |
|  |  |  |
|  |  |  |
|  |  |  |
|  |  |  |
|  |  |  |
|  |  |  |
|  |  |  |

**Web of Science**

Search date and time: 25/3/2024, 3:54pm: Retrieved 3 results

| S.No | Query | Records retrieved |
| --- | --- | --- |
| 1 | ALL=(adult OR “young adult” OR “adult children” OR aged) | 5814475 |
| 2 | ALL=(“sleep disorders” OR “sleep deprivation” OR “sleep disorders, intrinsic” OR “sleep  arousal disorders” OR “sleep apnea obstructive” OR “sleep wake disorders”) | 48212 |
| 3 | ALL=(periodontitis OR “chronic periodontitis” OR “aggressive periodontitis” OR periodont*) | 130514 |
| 4 | #1 AND #2 AND #3 | 25 |
| 5 | #1 AND #2 AND #3 and Review Article (Document Types) | 3 |
