# Supplementary material for: Association between sleep and periodontal disease in adults—an umbrella review
Source: Front Oral Health. 2026 Mar 12;7:1761243. doi: 10.3389/froh.2026.1761243 (PMC13018118; doi:10.3389/froh.2026.1761243)
Supplement: Supplementary file 2 [file Datasheet1.pdf]

Visual Abstract

Association between Sleep Quality & Periodontal Disease in Adults: An Umbrella Review

AMRITA VISHWA VIDYAPEETHAM

Objective: To assess the association of quality of sleep and occurrence of periodontal disease in adults

Methods

Population — Adults

891427 participants aged ≥18 years

Study design — Umbrella Review

Databases Searched

- PubMed
- ProQuest
- CINAHL (Available via EBSCOhost)
- Cochrane Library
- Scopus
- Clarivate Web of Science

Results

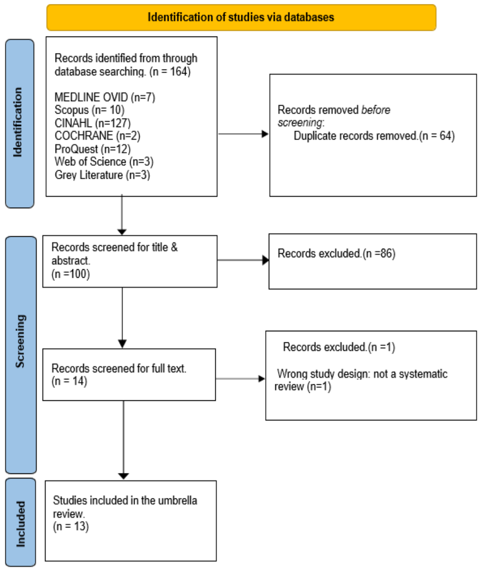

Appraisal of Methodological Quality of the included Systematic Reviews

JBI Critical Appraisal items	1	2	3	4	5	6	7	8	9	10	11	Overall Score
Lembo 2021	Yes	Yes	No	Yes	No	Yes	Yes	No	No	No	Yes	23
Wu 2022	Yes	Yes	No	Yes	Yes	Yes	Yes	Yes	Yes	Yes	Yes	31
Qun Zhou 2024	Unclear	Unclear	No	Yes	Yes	Yes	Yes	Yes	Yes	Yes	Yes	29
Edoardo Bianchi 2022	Yes	Yes	Yes	No	Yes	Yes	Yes	Yes	No	Yes	Yes	28
Giorgio Bianchi 2022	Yes	Unclear	No	Yes	Yes	Yes	Yes	Unclear	No	Yes	Unclear	26
Khodadadi 2022	Unclear	Yes	No	Yes	No	Yes	Yes	Yes	Yes	Yes	Yes	28
Malaiappan 2019	Unclear	No	No	No	No	Unclear	Yes	No	No	No	Yes	17
Al-Jewair 2015	Yes	Yes	Yes	Yes	No	Yes	Yes	Yes	No	Unclear	Yes	28
Schmidilin 2020	Yes	Yes	No	Yes	No	Yes	Yes	Yes	No	Unclear	Yes	26
Liu 2022	Unclear	Yes	Yes	Yes	No	Yes	Yes	Yes	Yes	Unclear	Yes	29
Muniz 2021	Unclear	Yes	No	Yes	Unclear	Yes	Yes	Unclear	No	Yes	Yes	26
Molina 2022	Yes	Yes	Yes	Yes	Yes	Yes	Yes	Yes	Yes	Unclear	Yes	32
Feixiang Zhou 2021	Unclear	Yes	Yes	Yes	Yes	Yes	Yes	Yes	Yes	Yes	Yes	32

● = Yes ● = No ● = Unclear Scoring: Yes= 3, Unclear=2 , No=1

Traffic Stop Light

Systematic Review	Number of Studies	Number of Participants	Odd's Ratio	Upper Limit	Lower Limit	GRADE	Heterogeneity	Stoplight
**Wu 2022**	6	1,07,777	1.19	1.16	1.23	Not assessed	0%	Green
**Zhou 2024a** -Short sleep Duration	10	66,516	1.04	0.83	1.29	Not assessed	94.4%	Red
**Zhou 2024b -** Long Sleep Duration	8	64,449	1.12	0.94	1.33	Not assessed	38.6%	Red
**Khodadadi 2022**	10	30,994	2.17	1.66	2.83	Not assessed	43%	Green
**Al-Jewair 2015**	4	29,955	1.65	1.11	2.46	Not assessed	92%	Green
**Liu 2022**	10	43,296	1.83	1.52	2.2	Not assessed	40%	Green
**Molina 2022**	6	43,342	1.65	1.21	2.25	Not assessed	86.5%	Green
**Feixiang Zhou 2021**	7	40,196	1.06	0.89	1.25	Not assessed	72.8%	Red

Association between sleep quality and periodontal disease	Green
No Association between sleep quality and periodontal disease	Red

Summary

Positive association between **poor sleep quality** and **periodontal disease.**

Outcome

Periodontitis

Sleep quality

Exposure

Dr. Keerthana Rajeev
MDS Resident,
Dept of Public Health Dentistry
Amrita School of Dentistry, Kochi
